# Hydration of Camphene over PW-SBA-15-SO_3_H

**DOI:** 10.3390/molecules28010006

**Published:** 2022-12-20

**Authors:** José Castanheiro

**Affiliations:** MED, DQB, Escola de Ciências e Tecnologia, Universidade de Évora, 7006-554 Évora, Portugal; jefc@uevora.pt

**Keywords:** camphene, hydration, SBA-15, heteropolyacids, sulfonic acid groups

## Abstract

The hydration of camphene was carried out over SBA-15 with sulfonic acid groups and tungstophosphoric acid at 50 °C. The main product of camphene hydration was isoborneol, with camphene hydrate and borneol as byproducts. The catalytic activity increased with the amount of tungstophosforic acid (PW) immobilized on the silica support until a maximum, which was obtained with the PW4-SBA-15-SO_3_H material (16.4 wt.%). When the amount of PW immobilized on SBA-15 increased (PW5-SBA-15-SO_3_H, 21.2 wt.%), the catalytic activity decreased. The catalytic activity of PW4-SBA-15-SO_3_H increased with the water content of the solvent, until a maximum was reached with 50% water. With higher water concentrations, a decrease in the catalytic activity was observed. The selectivity to isoborneol was 90% at 99% camphene conversion in the presence of the PW4-SBA-15-SO_3_H catalyst. The catalytic stability of the PW4-SBA-15-SO_3_H material during camphene hydration was studied by performing consecutive batch runs with the same catalyst sample. After the third run, a trend towards stabilized catalytic activity was observed. A kinetic model is also proposed.

## 1. Introduction

**Figure 1:**
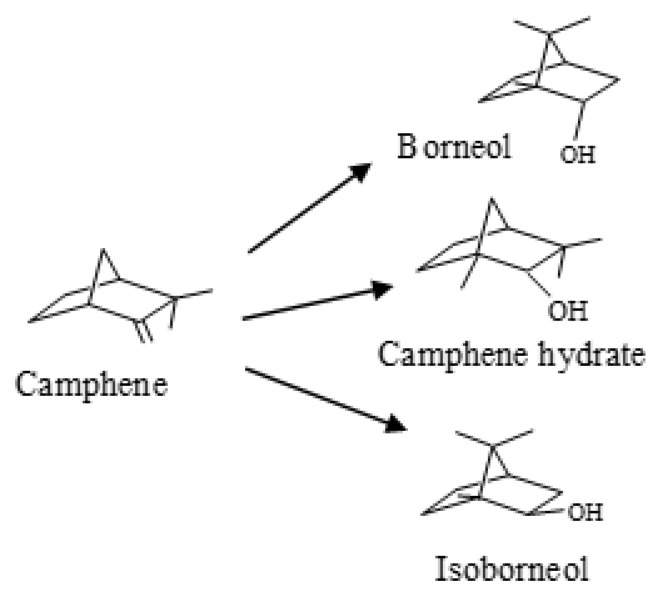
Scheme of camphene hydration.

The hydration of terpenes is an important synthesis route to obtain valuable compounds with many applications in the perfumery and pharmaceutical industries [1,2,3,4,5]. Isoborneol and borneol obtained by camphene hydration have applications in the formulation of soaps, cosmetic perfumes, and medicines [1,2]. Figure 1 shows the scheme of camphene hydration.

Isoborneol is also an intermediate in the synthesis of camphor [6]. Camphene hydration is carried out using homogeneous catalysts, such as HClO_4_ [7], H_4_SiW_12_O_40_ [8], and H_3_PW_12_O_40_ [8]. The homogenous catalysts have some environmental problems and economic inconveniences. For example, it is difficult to remove them from reaction mixtures and their reutilization becomes difficult. In order to overcome these problems, homogenous catalysts have been replaced by heterogeneous catalysts. The heterogeneous catalysts are more easily separated from reaction mixtures and can be reused [9,10,11,12].

Camphene hydration was performed using USY zeolite [13]. Materials were prepared with different extra-framework aluminum species. Valente et al. [13] observed that the parent HY or HY with low dealumination did not have catalytic activity, due to the surfaces of HY zeolite or low dealuminated zeolite being very hydrophilic. The solvent inside the porous system was richer in water content than the bulk solution. Camphene molecules had some difficulty in accessing the active site due to the layers of water molecules. The catalytic activity increased with increasing degree of dealumination, which was explained by the increase in the hydrophobicity of USY zeolite [13]. The selectivity to the isoborneol was approximately 90% at near complete camphene conversion.

In a previous work, SBA-15 with sulfonic acid groups was used as a catalyst in the conversion of pinene [14]. This mesostructured silica was used to immobilized PW [15,16,17]. n-Decane hydroisomerization [15], the conversion of glycerol to acrolein [16], the ethoxylation of terpenes [17] and the esterification of fatty acids [17] have been performed using PW immobilized on SBA-15.

In this work, we report the hydration of camphene over PW-SBA-15-SO_3_H. In order to optimize the amount of PW immobilized on SBA-15, different materials were prepared. The effect of solvent composition, amount of catalyst, and initial camphene concentration were studied. The catalytic stability of the PW4-SBA-15-SO_3_H material was also studied. A kinetic model was proposed.

## 2. Results and Discussion

### 2.1. Catalyst Characterization

**Figure 2:**
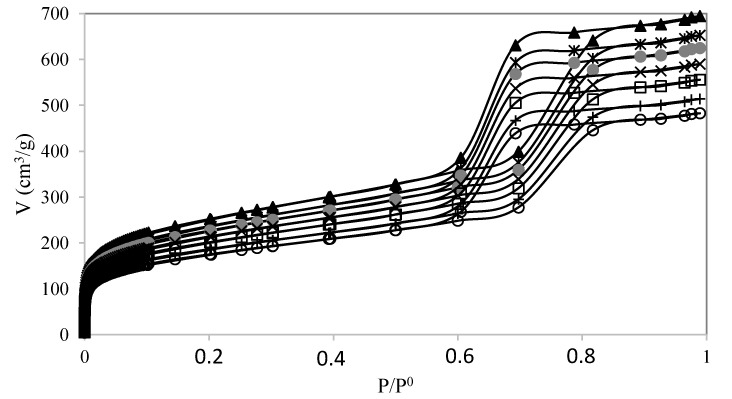
N_2_ isotherms of materials. (▲) SBA-15; (∗) SBA-15-SO_3_H; (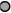) PW1-SBA-15-SO_3_H; (×) PW2-SBA-15-SO_3_H; (□) PW3-SBA-15-SO_3_H; (+) PW4-SBA-15-SO_3_H; (○) PW5-SBA-15-SO_3_H.

N_2_ isotherms at 77 K of the catalysts are shown in Figure 2. All catalysts displayed isotherms characteristic of the mesostructured SBA-15 material. The surface area (A_BET_) and porous volume (Vp) of the PW-SBA-15-SO_3_H catalyst are shown in Table 1. The A_BET_ and V_P_ diminished when the quantity of the PW immobilized on SBA-15 increased, which may be due to the reduction in the surface area to N_2_ adsorption. In previous studies, the A_BET_ and Vp decreased with the amount of PW immobilized on the mesostructured silica [15,16,17]. According to Gagea et al. [15], the heteropolyacid anions (H_2_PW_12_O_40_^−^) were trapped inside the SBA-15 particles. The PW molecules stayed fixed on the pore walls.

Table 1 shows the acidity of the materials. When the amount of PW on SBA-15 increased, the acidity of the materials also increased, which may be due to the increased amount of H^+^ with PW loading of the SBA-15 material [15,16].

**Figure 3:**
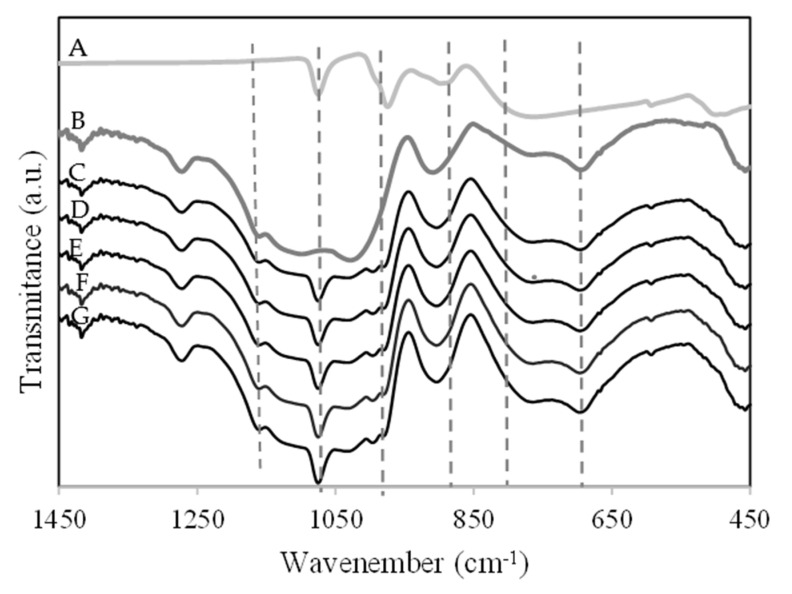
ATR-FTIR spectra of the catalysts: (**A**) PW; (**B**) SBA-15-SO_3_H; (**C**) PW1-SBA-15-SO_3_H, (**D**) PW2-SBA-15-SO_3_H, (**E**) PW3-SBA-15-SO_3_H, (**F**) PW4-SBA-15-SO_3_H, (**G**) PW5-SBA-15-SO_3_H.

Figure 3 shows the ATR-FTIR spectra of PW (A), SBA-15-SO_3_H (B), PW1-SBA-15-SO_3_H (C), PW2-SBA-15-SO_3_H (D), PW3-SBA-15-SO_3_H (E), PW4-SBA-15-SO_3_H (F), and PW5-SBA-15-SO_3_H (G). The PW material exhibited principal IR bands at 1080, 985, 890, and 839 cm^−1^, which were attributed to the ν_as_ P-O_a_, W = O_t_, W-O_c_-W, and W-O_e_-W of the Keggin structure of PW, respectively [16,18]. Additionally, the peaks from the -SO_3_H group were at 530, 620, 1068, and 1190 cm^−1^ [19]. The main bands of PW were present in the ATR-FTIR spectrum of PW-SBA-15-SO_3_H (Figure 3). However, some bands characteristic of Keggin units were overlapped with the bands of the SBA-15. In a previous work [15,16,17], when PW was supported on SBA-15, some major bands were also not observed. The peak from the -SO_3_H group at 1190 cm^−1^ was present in the ATR-FTIR spectrum of the PW-SBA-15-SO_3_H material (Figure 3). In addition, some peaks of the -SO_3_H group were overlapped with the bands of mesostructured silica SBA-15 [19].

**Figure 4:**
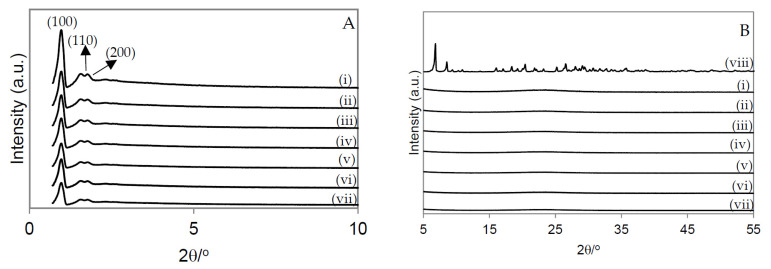
X-ray diffractograms of the materials. (**A**) 2θ region of 0.7° to 10.0°; (**B**) 2θ region of 5.0° to 55.0°; (i) SBA-15; (ii) SBA-15-SO_3_H; (iii) PW1-SBA-15-SO_3_H; (iv) PW2-SBA-15-SO_3_H; (v) PW3-SBA-15-SO_3_H; (vi) PW4-SBA-15-SO_3_H; (vii) PW5-SBA-15-SO_3_H; (viii) PW.

Figure 4A shows the XRD spectra of the catalysts. SBA-15 shows three diffraction peaks (100), (110), and (200), which corresponded to the two-dimension hexagonal mesostructure. All catalysts with PW immobilized on SBA-15-SO_3_H displayed the diffraction peak at the 2θ region, which indicated that the structure of SBA-15 was preserved after the immobilization of PW and the sulfonic acid groups onto silica [15,16].

Figure 4B displays the XRD spectra of the catalysts at the 2θ region of 5° to 55°. The peaks characteristic of PW (Figure 3B-(viii)) did not appear on the XRD spectrum of the SBA-15-SO_3_H material, which suggested that the PW units were very well dispersed [16,17].

**Figure 5:**
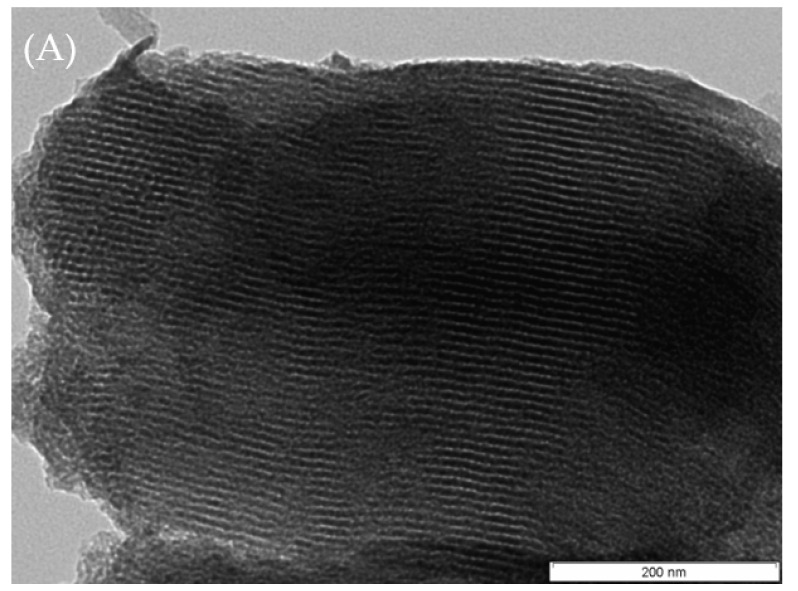

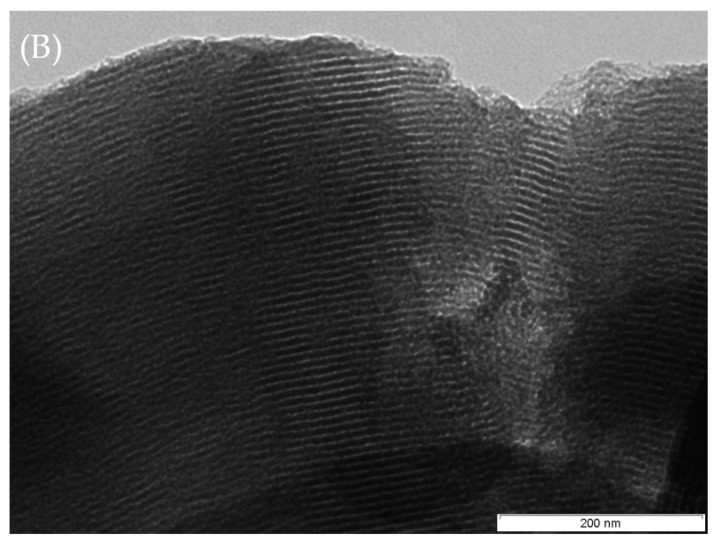
TEM images: (**A**) SBA-15; (**B**) PW4-SBA-15-SO_3_H.

Figure 5 shows the TEM images of the SBA-15 (Figure 5A) and PW4-SBA-15-SO_3_H materials (Figure 5B). The immobilization of PW and the introduction of the sulfonic acid groups did not seem to affect the porous system of the SBA-15 [15,16,17].

### 2.2. Catalytic Experiments

**Figure 6:**
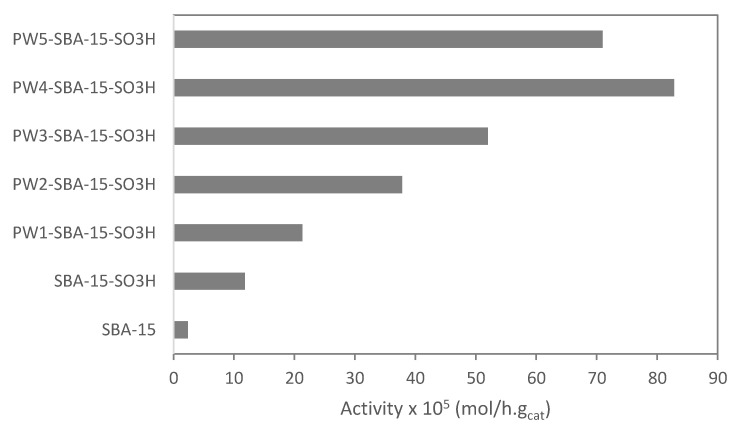
Camphene hydration over PW-SBA-15-SO_3_H catalysts. Initial activity of the materials. Reaction conditions: T = 50 °C; m_cat_ = 0.482 g; V = 114 mL of aqueous acetone (1:1, V/V); n_camphene_ = 7.5 mmol, t = 4 h.

Figure 6 shows the initial activity of SBA-15, SBA-15-SO_3_H, PW1-SBA-15-SO_3_H, PW2-SBA-15-SO_3_H, PW3-SBA-15-SO_3_H, PW4-SBA-15-SO_3_H, and PW5-SBA-15-SO_3_H. The catalytic activity of the materials increased with the amount of PW immobilized on SBA-15-SO_3_H until a maximum. This behavior can be explained by the increased acidity of the mesostructured silica and the amount of W species [17,20]. The number of active sites on SBA-15 may have been increased (Table 1). However, at a high amount of PW immobilized on SBA-15 (sample PW5-SBA-15-SO_3_H catalyst), the catalytic activity decreased. This behavior may be due to the existence of some internal diffusion limitations inside the SBA-15 material [16,17]. The total porous volume and surface area (A_BET_) decreased with the amount of PW on SBA-15 (Table 1). It is important to note that products were not observed on the surface of catalyst. Additionally, no oligomerization of camphene occurred under these reaction conditions.

**Figure 7:**
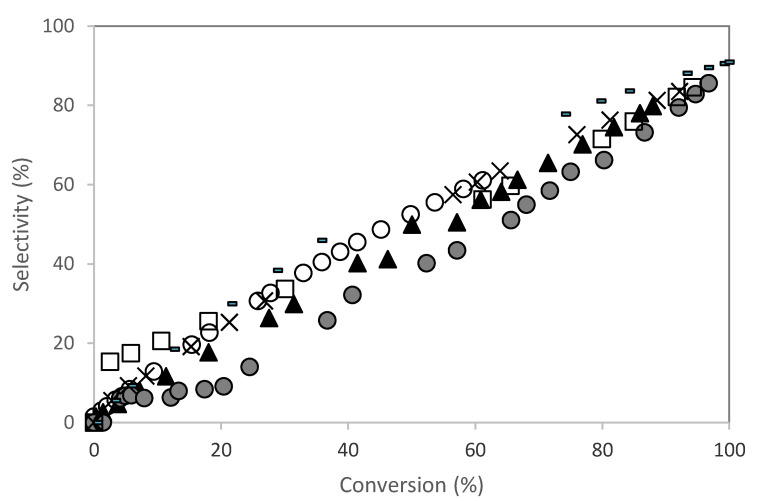
Camphene hydration over PW-SBA-15-SO_3_H catalysts. Isoborneol selectivity (%) versus camphene conversion. (○) SBA-15-SO_3_H; (▲) PW1- SBA-15-SO_3_H; (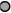) PW2-SBA-15-SO_3_H; (×) PW3-SBA-15-SO_3_H; (-) PW4-SBA-15-SO_3_H; (□) PW5-SBA-15-SO_3_H. Reaction conditions: T = 50 °C; m_cat_ = 0.482 g; V = 114 mL of aqueous acetone (1:1, V/V); n_camphene_ = 7.5 mmol.

Figure 7 shows the selectivity to isoborneol. All catalysts showed high selectivity to the isoborneol compound. Apparently, the selectivity to isoborneol was not affected by the change in the acidity of the materials. According to Valente et al. [13], the selectivity to isoborneol increased slightly with the amount of acid sites. However, in this work, a relationship was not observed between the selectivity to isoborneol and the acidity of the materials.

**Figure 8:**
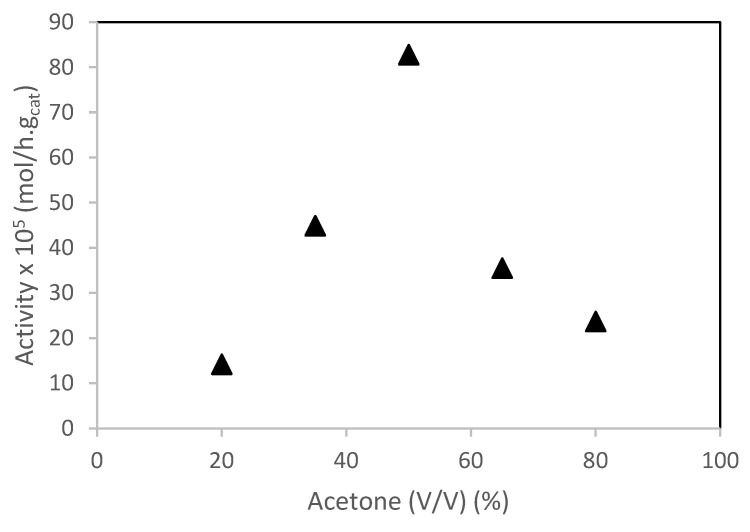
Hydration of camphene over PW4-SBA-15-SO_3_H catalysts. The effect of the solvent. Reaction conditions: T = 50 °C; m_cat_ = 0.482 g; V = 114 mL of aqueous acetone; n_camphene_ = 7.5 mmol, t = 4 h.

The effect of the solvent (aqueous acetone) on camphene hydration using the PW4-SBA-15-SO_3_H catalyst was studied. Figure 8 shows the catalytic activity of the PW4-SBA-15-SO3H material versus acetone (%). The results can be explained as follows:

- at low water content (high amount of acetone), the catalytic activity increased with increasing water content. This behavior may be due to low amount of water inside the PW4-SBA-15-SO_3_H surface. When the amount of water increased, the catalytic activity increased as well, until a maximum was reached at 50% of water.
- at high water content (low amount of acetone), it is expected that the solvent inside the PW4-SBA-15-SO_3_H pore system was richer in water content than the bulk solution. The layer of water molecules surrounding the active sites form a barrier hindering the diffusion of camphene. Consequently, the camphene sorption coefficient as well as the activity, decreased.

**Figure 9:**
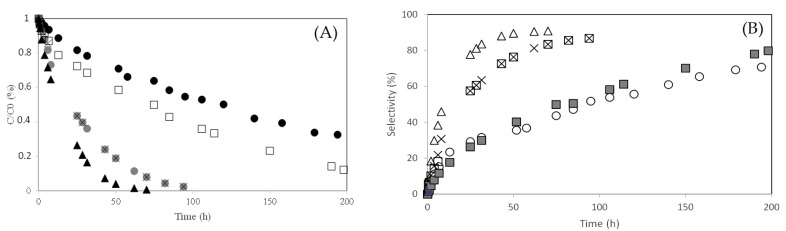
Hydration of camphene over PW4-SBA-15-SO_3_H catalysts. The effect solvent. (**A**) [camphene]/[Camphene]_0_ *versus* time (h): (⚫) 20% of acetone; (×) 35% of acetane; (▲) 50% of acetone; (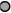) 65% acetone; (□) 80% of acetone; (**B**) Isoborneol selectivity (%): (○) 20% of acetone; (×) 35% of acetone; (△) 50% of acetone; (□) 65% of acetone; (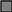) 80% of acetone. Reaction conditions: T = 50 °C; m_cat_ = 0.482 g; V = 114 mL of aqueous acetone; n_camphene_ = 7.5 mmol.

Figure 9A shows the effect of the solvent on the camphene profile. The selectivity of the PW4-SBA-15-SO_3_H catalyst to isoborneol increased with the amount of water in the reaction mixture (Figure 9B). This behavior may be explained by the increased amount of water molecules inside the pores of the catalyst. The maximum selectivity to isoborneol (90%) was obtained with a 50:50 (V/V) mixture of acetone:water. When the amount of water increased above the 50%, the selectivity to isoborneol decreased. This behavior may be due to a higher amount of water inside the pores of the catalyst and, consequently, the concentration of camphene near the active site was low.

#### 2.2.1. Effect of the catalyst amount

**Figure 10:**
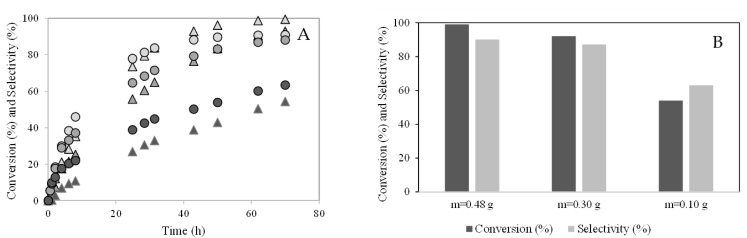
Hydration of camphene over PW4-SBA-15-SO_3_H catalyst. Effect of the catalyst amount. (**A**) Camphene conversion (%) versus time (h): (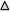) m = 0.48 g; (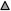) m = 0.30 g; (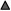) m = 0.10 g; Isoborneol selectivity (%): (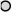) m = 0.48 g; (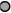) m = 0.30 g; (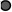) m = 0.10 g. (**B**) Camphene conversion (%) and selectivity (%) to the isoborneol at 70 h of reaction.

The effect of the amount of PW4-SBA-15-SO_3_H on camphene conversion was studied. The initial concentration of camphene (C = 0.065 mol.dm^−3^) and the reaction temperature (T = 50 °C) were kept constant. Figure 10A shows the effect of the amount of PW4-SBA-15-SO_3_H on camphene conversion and isoborneol selectivity. The camphene conversion increased with the amount of catalyst due to the increased number of active sites present in the reaction mixture [21]. The isoborneol selectivity increased with the amount of catalyst. However, when the amount of catalyst used increased from 0.30 to 0.48 g, the selectivity to isoborneol did not increase, which may be due to the existence of a mass transfer limitation when excess catalyst was used under the same reaction conditions [21]. Figure 10B shows camphene conversion (%) represented by dark bars and selectivity (%) to isoborneol represented by light bars after 70 h of reaction. The selectivity to isoborneol decreased when the camphene conversion also decreased. This behavior may be explained by isoborneol being a product obtained from camphene and camphene hydrate compounds (kinetic model proposed).

#### 2.2.2. Effect of the Initial Concentration of Camphene

**Figure 11:**
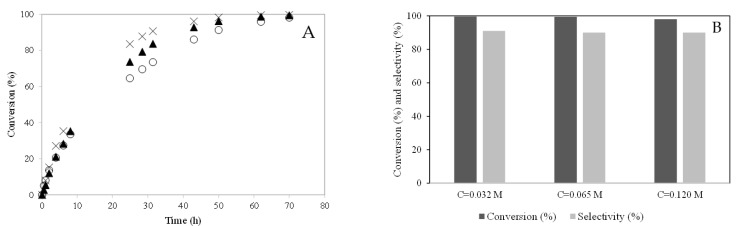
Hydration of camphene over PW4-SBA-15-SO_3_H catalyst. Effect of the initial concentration of camphene. (**A**) conversion (%) versus time (h): (×) C = 0.032 mol.L^−1^; (▲) C = 0.065 mol.L^−1^; (○) C = 0.12 mol.L^−1^. (**B**) Camphene conversion (%) and selectivity (%) to the isoborneol at 70 h of reaction.

The initial concentration of camphene was also studied. The temperature (T = 50 °C) and the amount of catalyst (m = 0.48 g) were kept constant. Figure 11A shows the effect of the initial concentration of camphene on the conversion of this terpene. The initial reaction rate increased slightly when the initial camphene concentration decreased, which may be explained by the low quantity of camphene molecules for the same amount catalyst. Figure 11B shows camphene conversion (%) represented by dark bars and selectivity (%) to isoborneol represented by light bars after 70 h of reaction. After 70 h of reaction, the camphene conversion was quite similar (Figure 11B). The selectivity to isoborneol decreased slightly.

**Figure 12:**
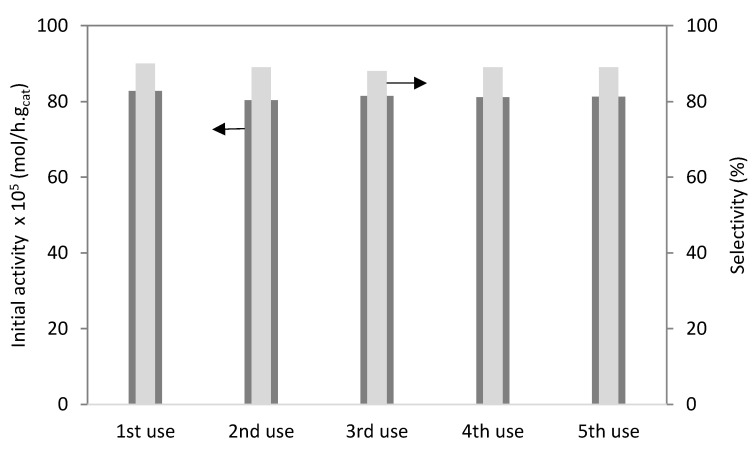
Hydration of camphene over PW4-SBA-15-SO_3_H. Catalytic stability (initial activity) and isoborneol selectivity (%) after 70 h.

Figure 12 displays the catalytic activity of the PW4-SBA-15-SO_3_H material. The catalyst showed good activity after five uses. After the reaction, the PW4-SBA-15-SO_3_H material was characterized by ICP. A 3% loss of PW occurred. Products were not observed on the surface of the catalyst. Additionally, no oligomerization of camphene occurred under these reaction conditions. The lost PW may be due to some species being adsorbed on the SBA-15 surface. PW existed in the pore wall of SBA-15 [15]. Selectivity to isoborneol was similar (about 90%) after five utilizations of the PW4-SBA-15-SO_3_H catalyst.

### 2.3. Kinetic Modeling

**Figure 13:**
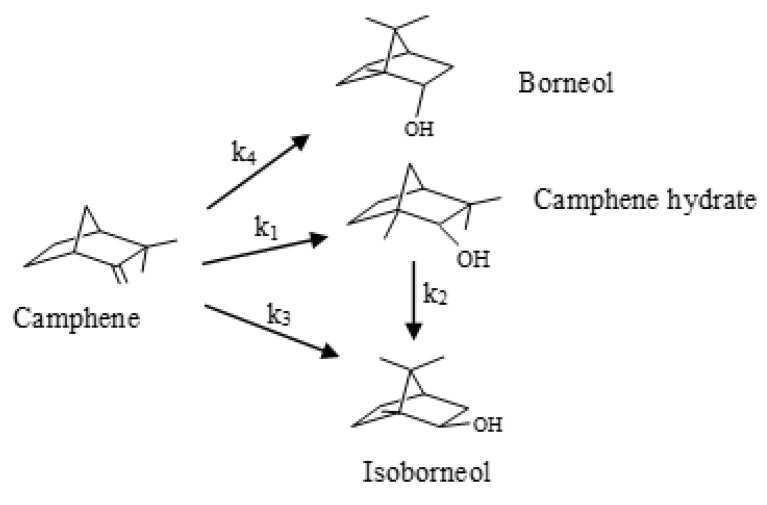
Reaction network used for kinetic modelling in camphene hydration.

The Langmuir–Hinshelwood (LH) mechanism has been widely used in the kinetic study of heterogeneous catalytic systems. Based on the surface reaction between two adsorbed species, the LH mechanism forecasted the kinetic data very well in the hydration of cyclohexene over ion-exchange resin and H-ZSM-5 [21], the hydrolysis of ethyl benzoate [22], the liquid-phase dimerization of isoamylenes [23], the liquid-phase hydrogenation of cinnamaldehyde [24], the esterification of lactic acid with ethanol [25], the esterification of propanol with ethanoic acid [26], the synthesis of tert-amyl methyl ether [27], and the catalytic hydrogenation of d-lactose to lactitol [28]. A kinetic model was proposed assuming a Langmuir-Hinshelwood mechanism, where the reaction is controlled by the surface reaction step. Additionally, it was assumed that the camphene and camphene hydrate adsorbed on the active sites, while the water, isoborneol, and other products did not adsorb on the active sites. The proposed reaction scheme for camphene hydration is shown in Figure 13. The variables of the model are: camphene concentration ([C]), camphene hydrate concentration ([HC]), isoborneol concentration ([I]), and the concentration of the other compounds ([O]). K_C_ and K_HC_ are the adsorption equilibrium constant of the camphene and camphene hydrate, respectively.

The batch reactor was operated under isothermal and isobaric conditions.

The reaction rates are given by:

(1) $$r_{1}=\frac{k_{1}\times \left[C\right]\times K_{C}}{1+K_{C}\times \left[C\right]+K_{HC}\times \left[HC\right]}$$

(2) $$r_{2}=\frac{k_{2}\times \left[HC\right]\times K_{HC}}{1+K_{C}\times \left[C\right]+K_{HC}\times \left[HC\right]}$$

(3) $$r_{3}=\frac{k_{3}\times \left[C\right]\times K_{C}}{1+K_{C}\times \left[C\right]+K_{HC}\times \left[HC\right]}$$

(4) $$r_{4}=\frac{k_{4}\times \left[C\right]\times K_{C}}{1+K_{C}\times \left[C\right]+K_{HC}\times \left[HC\right]}$$

where *k*_1_, *k*_2_, *k*_3_ and *k*_4_ are kinetic reaction constants, *C* represents camphene, and *HC* represents the camphene hydrate.

The molar balance in the batch reactor is given by:

(5) $$\frac{V}{W}\times \frac{d\left[C\right]}{dt}=-r_{1}-r_{3}-r_{4}$$

(6) $$\frac{V}{W}\times \frac{d\left[HC\right]}{dt}=+r_{1}-r_{2}$$

(7) $$\frac{V}{W}\times \frac{d\left[I\right]}{dt}=+r_{2}+r_{3}$$

(8) $$\frac{V}{W}\times \frac{d\left[O\right]}{dt}=+r_{4}$$

where the *C* represents camphene, *HC* represents the camphene hydrate, *I* represents isoborneol, and *O* represents “other molecules”.

The optimization was performed using the SOLVER routine in a Microsoft Excel spreadsheet.

**Figure 14:**
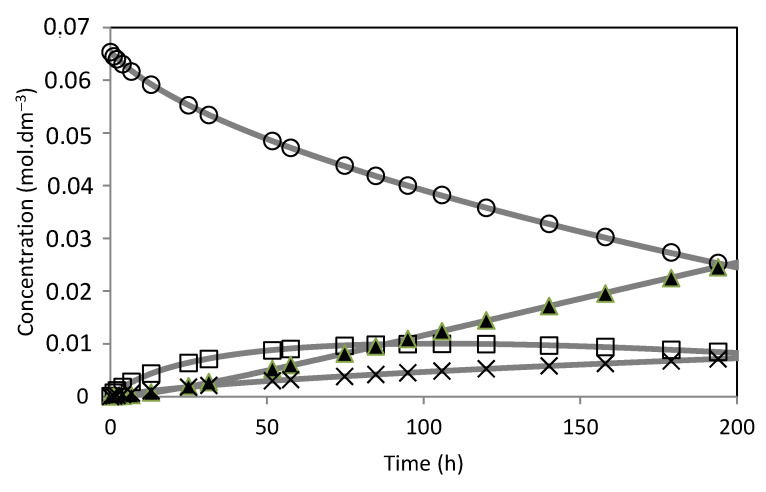
Camphene hydration over SBA-15-SO_3_H catalyst. (○) Camphene; (□) Camphene hydrate; (▲) Isoborneol; (×) Others. The lines represent the model fitted to the experimental points.

**Figure 15:**
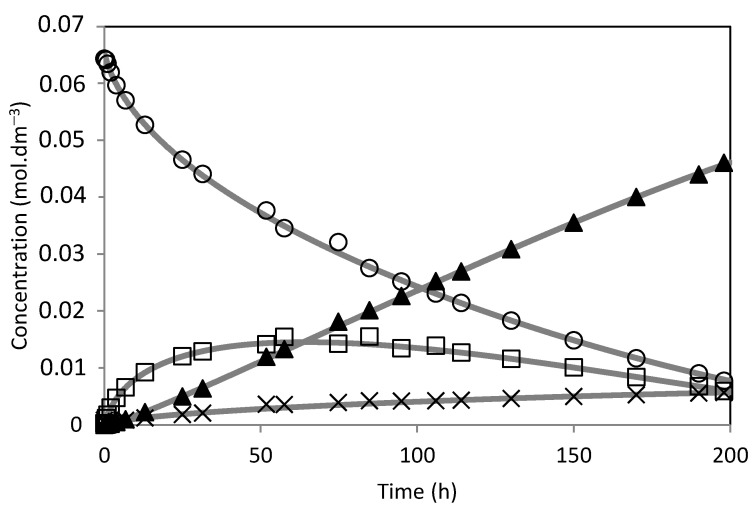
Camphene hydration over PW1-SBA-15-SO_3_H catalyst. (○) Camphene; (□) Camphene hydrate; (▲) Isoborneol; (×) Others. The lines represent the model fitted to the experimental points.

**Figure 16:**
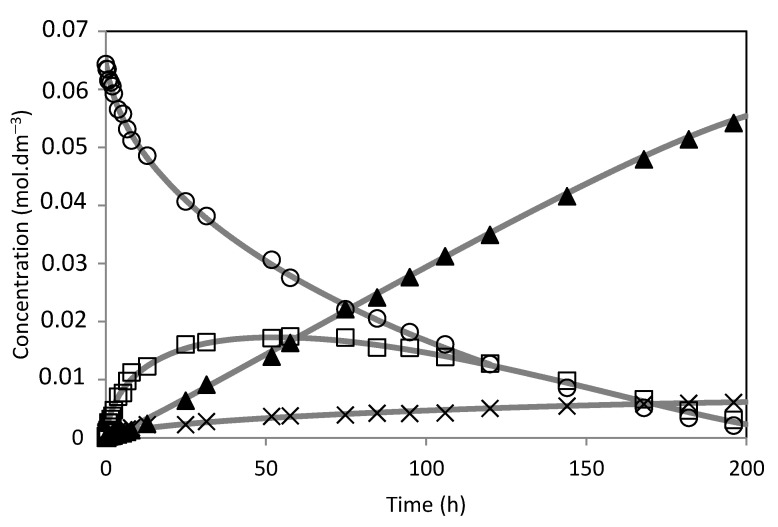
Camphene hydration over PW2-SBA-15-SO_3_H catalyst. (○) Camphene; (□) Camphene hydrate; (▲) Isoborneol; (×) Others. The lines represent the model fitted to the experimental points.

**Figure 17:**
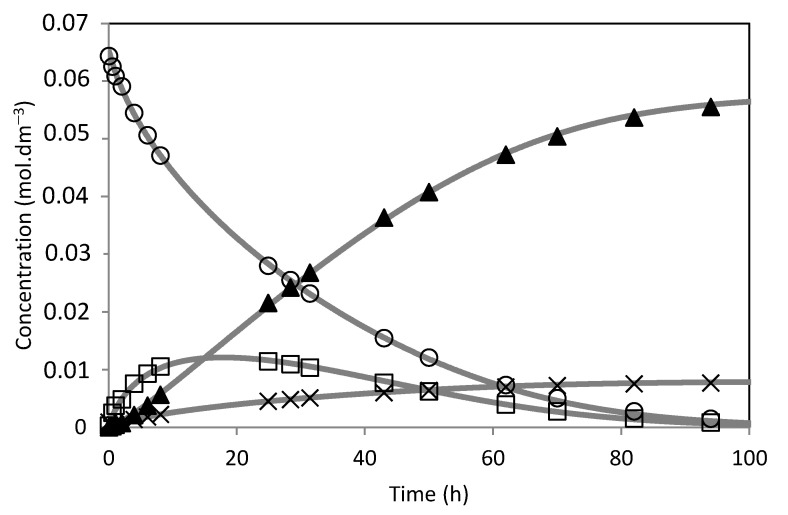
Camphene hydration over PW3-SBA-15-SO_3_H catalyst. (○) Camphene; (□) Camphene hydrate; (▲) Isoborneol; (×) Others. The lines represent the model fitted to the experimental points.

**Figure 18:**
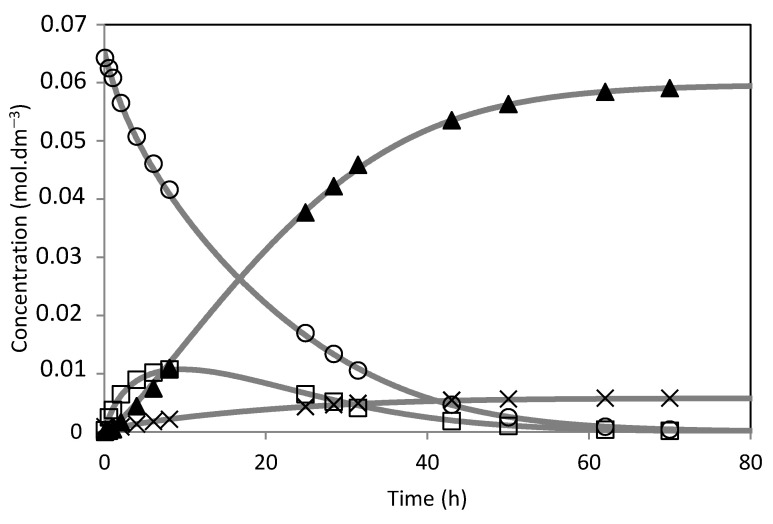
Camphene hydration over PW4-SBA-15-SO_3_H catalyst. (○) Camphene; (□) Camphene hydrate; (▲) Isoborneol; (×) Others. The lines represent the model fitted to the experimental points.

**Figure 19:**
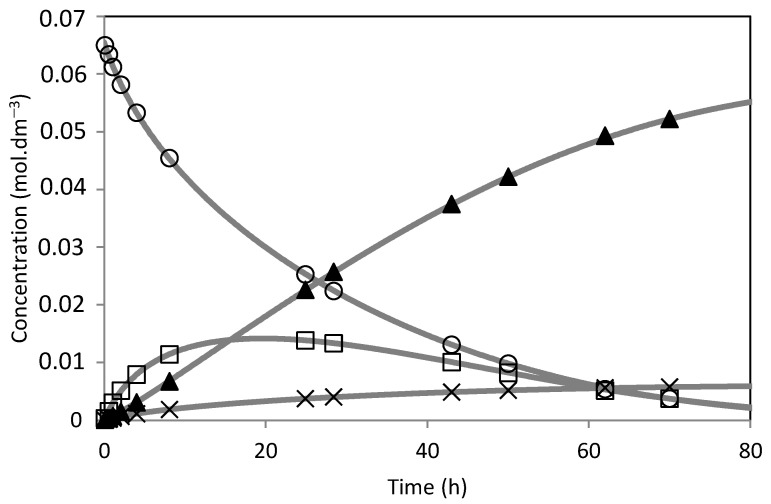
Camphene hydration over PW5-SBA-15-SO_3_H catalyst. (○) Camphene; (□) Camphene hydrate; (▲) Isoborneol; (×) Others. The lines represent the model fitted to the experimental points.

The model was fitted to the experimental results (Figure 14, Figure 15, Figure 16, Figure 17, Figure 18 and Figure 19). The solid lines represented the kinetic model fitted to the experimental data. The kinetic model fit the experimental concentration data quite well.

Table 2 shows the model parameters obtained by application of the kinetic model to the experimental data. The kinetic constants increased with the acidity and amount of PW immobilized on SBA-15-SO_3_H (Table 1). The adsorption constant of camphene and the camphene hydrate tended to decrease with the amount of PW on the material. There were some changes in the hydrophobic/hydrophilic balance on the catalyst’s surface.

Table 3 shows the activity and selectivity to isoborneol of different materials used for camphene hydration. The catalytic activity of PW4-SBA-15-SO_3_H for camphene hydration was found to be higher than the catalytic activity of USY zeolite.

## 3. Materials and Methods

### 3.1. Materials

Template (Pluronic P-123), 1-butanol (99.8%), camphene (98%), nonane (99%), tetraethylorthosilicate (TEOS), HCl (37%), tungstophosphoric acid, (3-mercaptopropyl) triethoxy-silane, hydrogen peroxide (30%), ethanol (96%), and acetone (99%) were acquired from Sigma–Aldrich.

### 3.2. Preparation of Materials

The preparation of PW-SBA-15-SO_3_H catalysts was carried out using a similar procedure as described by Yu et al. [29]. Pluronic P-123 (4 g) were dispersed in 144 mL of distilled H_2_O, and different amounts of tungstophosphoric acid and 7.9 g of 35% HCl were added to the mixture under stirring at 40 °C for 1 h. After complete dissolution, 4 g of 1-butanol was added. The mixture was stirred for 1 h. After this period, a mixture of 8.6 g of tetraethylorthosilicate (TEOS) with 0.4 g of (3-mercaptopropyl)triethoxysilane was added. The solution was maintained under stirring at 40 °C for 24 h. After this period, 1.6 g of hydrogen peroxide (30%) was added to the mixture. The mixture was placed in a closed autoclave and heated at 100 °C for 24 h. The white solid was filtered and dried at 100 °C for 24 h. Finally, the fine powder obtained was washed (ethanol and HCl mixture) to remove the template.

### 3.3. Materials Characterization

A Micromeritics ASAP 2010 instrument was used to obtain the N_2_ isotherms at 77 K.

The amount of PW on SBA-15 was evaluated by ICP.

The ATR-FTIR spectra were obtained using a Perkin Elmer Spectrum 100 FTIR spectrophotometer.

XRD patterns of PW, SBA-15, and PW-SBA-15-SO_3_H materials were obtained using a Rigaku Miniflex powder diffractometer.

TEM photos were executed on a Hitachi S-2400 instrument.

Acid-base titration was used to obtain the total acid density (mmol H^+^.g^−1^) of the materials. The acid-base titrations were carried out according to previous work [29].

### 3.4. Catalytic Experiments

The camphene hydration reactions were carried out in a jacketed batch reactor (200 mL) at 50 °C. In a typical hydration experiment, the reactor was loaded with 114 mL of aqueous acetone (1:1, V/V) and 0.482 g of the catalyst. The reactions were initiated by adding 7.5 mmol of camphene.

The material PW4-SBA-15-SO_3_H was reused several times.

Nonane was used as an internal standard.

The samples were removed from the reactor periodically. The samples were analyzed by GC using a Hewlett Packard instrument equipped with a 30 m × 0.25 mm DB-1 column. The injector temperature was 180 °C and the detector temperature was 300 °C. The oven temperature program was as follows: started at 80 °C (4 min), ramp at 6 °C min^−1^ to 126 °C, and ramp at 10 °C min^−1^ to 300 °C. The products were identified by GC-MS using a FISONS MD800 (Leicestershire, UK) with the same column and temperature conditions.

The initial activity was calculated by the expression:

$$Initial\;activity={\left(\frac{dC_{camphene}}{dt}\right)}_{0}\times \frac{V}{w}$$

where *V* is the volume, *W* is the amount of catalyst, and ${\left(\frac{dC_{camphene}}{dt}\right)}_{0}$ is the scope of the line obtained by the linear regression using the camphene concentration during the first 4 h of the reaction.

## 4. Conclusions

Camphene hydration was performed using SBA-15 with sulfonic acid groups and PW as a catalyst. Different catalysts with the same amount of sulfonic acid groups but different PW amounts (1.7 to 22.1 wt.%) in SBA-15 were produced. The PW4-SBA-15-SO_3_H material (16.4 wt.%) exhibited higher catalytic activity than other catalysts.

All the catalysts showed great selectivity to isoborneol.

The stability of the PW4-SBA-15-SO_3_H catalyst was studied. After the second use, the catalyst presented high activity. The selectivity to isoborneol was not affected.

A kinetic model was developed, which fit the experimental data relatively well.

## Figures and Tables

**Table 1:** Characterization of the materials.

Materials	Acidity ^a^ (mmol H^+^/g)	HPW Amount (wt%)	A_BET_ (m^2^/g)	V_T_ ^b^ (cm^3^/g)
SBA-15	-	-	880	0.82
SBA-15-SO_3_H	0.40	-	772	0.74
PW1-SBA-15-SO_3_H	0.52	1.7	723	0.71
PW2-SBA-15-SO_3_H	0.68	4.2	707	0.68
PW3-SBA-15-SO_3_H	0.78	8.6	687	0.65
PW4-SBA-15-SO_3_H	1.32	16.4	654	0.61
PW5-SBA-15-SO_3_H	1.53	22.1	583	0.56

^a^ The total acid density was measured by titration; ^b^ (p/p°) = 0.98.

**Table 2:** Model parameters obtained by fitting the model to experimental data.

Catalyst	k_1_ (mol/g.h)	k_2_ (mol/g.h)	k_3_ (mol/g.h)	k_4_ (mol/g.h)	K_C_ (dm^−3^/mol)	K_HC_ (dm^−3^/mol)
SBA-15-SO_3_H	0.0003026	0.0000510	0.0000006	0.0000654	11.1937638	253.3123137
PW1-SBA-15-SO_3_H	0.0062009	0.0000767	0.0000001	0.0006799	1.3138595	241.6666349
PW2-SBA-15-SO_3_H	0.0084667	0.0000841	0.0000002	0.0008956	2.1688618	368.2589676
PW3-SBA-15-SO_3_H	0.0150343	0.0003588	0.0010349	0.0022176	0.9024512	109.2452103
PW4-SBA-15-SO_3_H	0.0414648	0.0008294	0.0034500	0.0043307	0.4439438	78.2818538
PW5-SBA-15-SO_3_H	0,0284237	0.0003753	0.0050847	0.0034519	0.4343751	70.5933381

**Table 3:** Camphene hydration over heterogenous catalysts. Comparison of the results with literature data.

Catalyst	Time (h)	Conversion (%)	Selectivity (%) Isoborneol	Initial Activity (mol/h.g_cat_)	Reference
USY	50	95	90	4.0 × 10^−4^	[5]
PW4-SBA-15-SO_3_H	70	99	96	8.2 × 10^−4^	Present work

## Data Availability

The compound used for the catalysis and raw characterization data studies herein reported are available from the author upon request.

## Sample Availability

Samples of the compounds are available from the author.
